# Cumulative Resting Heart Rate Exposure and Risk of All-Cause Mortality: Results from the Kailuan Cohort Study

**DOI:** 10.1038/srep40212

**Published:** 2017-01-09

**Authors:** Quanhui Zhao, Haibin Li, Anxin Wang, Jin Guo, Junxing Yu, Yanxia Luo, Shuohua Chen, Lixin Tao, Yuqing Li, Aiping Li, Xiuhua Guo, Shouling Wu

**Affiliations:** 1 Graduate School, North China University of Science and Technology, Tangshan, China; 2 Department of Cardiology, Kailuan Hospital, North China University of Science and Technology, Tangshan, China; 3 Department of Epidemiology and Health Statistics, School of Public Health, Capital Medical University, Beijing, China; 4 Beijing Municipal Key Laboratory of Clinical Epidemiology, Capital Medical University, Beijing, China; 5 Department of Neurology, Beijing Tiantan Hospital, Capital Medical University, Beijing, China; 6 Department of Rheumatology, Kailuan Hospital, North China University of Science and Technology, Tangshan, China; 7 Department of Surgery, Kailuan Hospital, North China University of Science and Technology, Tangshan, China

## Abstract

The relationship between cumulative exposure to resting heart rate (cumRHR) and mortality remain unclear in the general population. In the Kailuan cohort study, resting heart rate (RHR) was repeatedly measured at baseline and at years 2 and 4 by electrocardiogram among 47,311 adults aged 48.70 ± 11.68. The cumRHR was defined as the summed average RHR between two consecutive examinations multiplied by the time interval between with two examinations [(beats/min) * year]. A higher RHR was defined as ≥80 beats/min, and the number of visits with a higher RHR was counted. During a median of 4.06 years of follow-up, a total of 1,025 participants died. After adjusting for major traditional cardiovascular risk factors and baseline RHR, the hazard ratio for the highest versus lowest quartile of cumRHR was 1.39 (95% CI: 1.07–1.81) for all-cause mortality. Each 1-SD increment in cumRHR was associated with a 37% (HR: 1.37, 95% CI: 1.23–1.52) increased risk of death and displayed a J-shaped relationship. Compared with no exposure, adults who had a higher RHR at all 3 study visits were associated with a 1.86-fold higher risk (95% CI: 1.33–2.61) of mortality. In summary, cumulative exposure to higher RHR is independently associated with an increased risk of mortality.

Observational epidemiological study findings consistent with clinical trial findings have found that an elevated resting heart rate (RHR) is significantly associated with cardiovascular disease (CVD) and all-cause mortality both in healthy populations^1^ and patients with chronic heart failure (CHF)^2^,^3^,^4^,^5^, atrial fibrillation (AF)^6^,^7^,^8^, ischaemic stroke^9^,^10^, acute myocardial infarction^11^, chronic obstructive pulmonary disease^12^ and type 2 diabetes mellitus^13^. Two recent meta-analyses have also suggested that an elevated RHR increases the risk of cardiovascular mortality in the general population^14^,^15^. However, most studies have only focused on the prognostic value of the baseline RHR measured at only a single point time. However, a few studies have investigated the relationship between repeated measurements of RHR and the risk of all-cause mortality by treating RHR as a time-dependent variable^16^, including the Cardiovascular Health Study^17^, ORBIT-AF Study^6^, the Framingham Heart Study^18^ and the Copenhagen City Heart Study^19^.

However, information on how cumulative exposure to higher RHR increases the risk of death remains unknown. Cumulative exposure of measurements could be quantified and may yield additional information because the duration and intensity of exposure could be considered together^20^. Zemaitis *et al*.^21^ demonstrated that cumulative exposure to higher systolic blood pressure (cumSBP) was associated with increased risk of urine albumin-to-creatinine ratio (UACR) progression among adults without type 2 diabetes. Kishi *et al*.^22^ found that cumulative blood pressure exposure in early adulthood was associated with left ventricular dysfunction in middle age. Nikpour *et al*.^23^ compared “summary measures” of cumulative exposure to total cholesterol (TC), systolic blood pressure (SBP), and diastolic blood pressure (DBP) in terms of the ability to quantify coronary artery disease risk in patients with systemic lupus erythematosus (SLE). This analytical framework—using repeated measurements over a period of several years to estimate the cumulative exposure to resting heart rate (cumRHR) for each person—has not yet been applied to evaluate the association between cumRHR and the risk of all-cause mortality in the general population. Further, the association between the number of visits with a higher RHR (≥80 beats/min^24^) with the risk of mortality has not been well described.

In the current analysis, we used data from the baseline to the third examination in the Kailuan cohort study to calculate the cumRHR and to explore the association between cumRHR, the number of visits with a higher RHR and any cause of death. We also test whether there were sex-related differences. We hypothesise that higher cumRHR and the number of visits with a higher RHR are associated with a higher risk of all-cause mortality.

## Results

### Baseline characteristics

**Figure 1:**
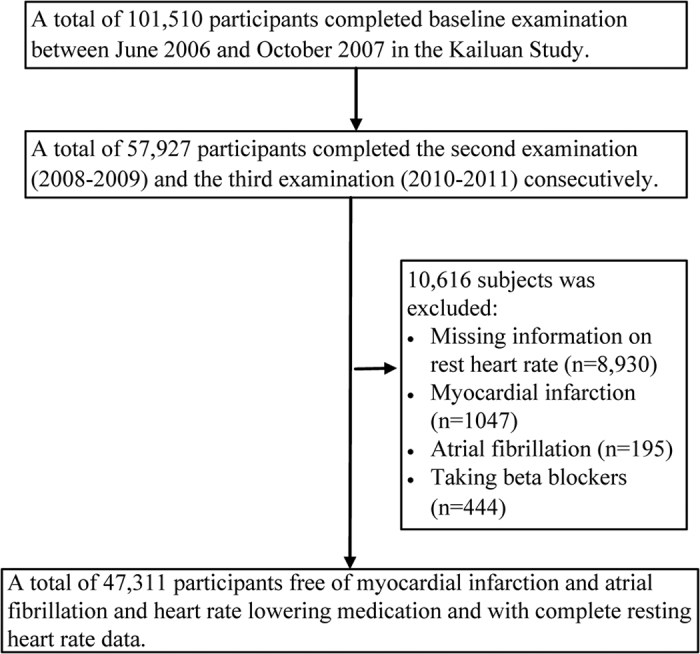
Flowchart of Kailuan cohort study.

Of a total of 101,510 individuals in the original cohort, 57,927 participants completed the second and third examinations consecutively. For the remaining participants, 1,686 participants who experienced myocardial infarction, atrial fibrillation or started taking beta blockers as well as 8,930 participants with missing information on RHR on any occasion from the baseline to the third examination were excluded (Fig. 1). Thus in the current analysis, 47,311 participants were included. The study population consisted of 78.2% men and 21.8% women, with a mean age ± standard deviation (SD) of 48.70 ± 11.68 years. The baseline characteristics of the 8,930 subjects with missing RHR data were different from the remaining 47,311 adults. The excluded participants were slightly older, more female, had a lower baseline systolic blood pressures, and had a lower proportion of smokers, alcohol users, hypertensives and diabetics (all *P* < 0.01) compared with the participants included in this study (Supplemental Table 1).

The average cumRHR ± SD was 298.66 ± 46.79 [(beats/min) * year], with a range from 156 to 698 [(beats/min) * year]. The baseline characteristics of the participants stratified by the quartile of cumRHR are summarized in Table 1. Participants in the highest quartile of cumRHR (Q4) were more likely to have a higher systolic BP, diastolic BP, BMI, fasting blood glucose, TC, and LDL-C, and they had a higher proportion of participants who engaged in physical activity, smoking, or drinking, or who had hypertension, diabetes mellitus, hyperlipidaemia, stroke or cancer compared with the participants in the lowest cumRHR quartile (Q1) (all *P* < 0.05, Table 1).

### Association with cumRHR

**Figure 2:**
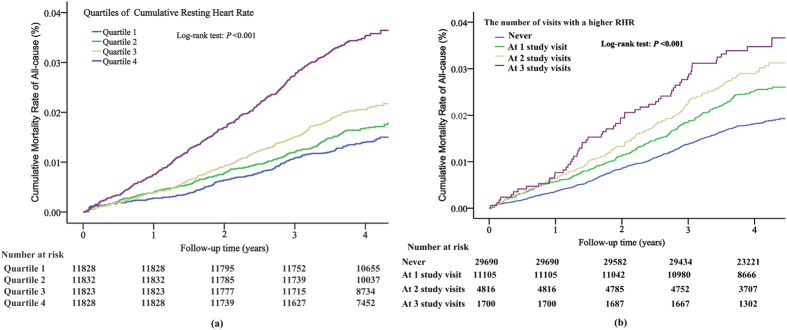
Cumulative mortality rate of all-cause according to quartile of cumRHR (**a**) and the number of visits with a higher RHR (**b**) estimating by Kaplan-Meier curve. cumRHR quartiles are as follows: Q1, <267.90; Q2, 267.90–292.84; Q3, 292.85–322.84; Q4, ≥322.85 (beats/min) * year. Higher RHR was defined as ≥80 beats/min. The number of visits with a higher RHR are as follows: never, at 1 study visit, at 2 study visits and at 3 study visits. Key: RHR, resting heat rate; CumRHR, cumulative resting heart rate exposure.

During a median of 4.06 years of follow-up from the third examination, a total of 1,025 (2.17%) participants died any of cause. The mortality rate was 54.44 per 10,000 person-years for the overall study population. The number of death events and risk of mortality in each quartile of cumRHR and the number of visits with a higher RHR are presented in Table 2. From the lowest cumRHR quartile to the highest, the mortality rate increased from 35.60 to 42.05, 52.94 and 90.91 per 10,000 person-years, respectively. Figure 2(a) shows the Kaplan-Meier mortality rate by the quartile of cumRHR. The log-rank test revealed that there was a significant difference between each quartile (χ^2^ = 149.69, *P* < 0.001). In the univariate Cox model, subjects in the highest cumRHR quartile had a higher mortality risk compared with the lowest quartile (unadjusted HR: 2.54, 95% CI: 2.12–3.04). After accounting for sociodemographic and cardiovascular risk factors, similar associations between the cumRHR categories and all-cause mortality were attenuated but remained statistically significant in model 3 (Table 2, adjusted HR:1.42, 95% CI: 1.13–1.78). Further adjustment for baseline RHR level did not substantially change the associations (model 4). Individuals in the highest cumRHR quartile had a 39% increased risk of all-cause mortality (Table 2, adjusted HR: 1.39, 95% CI: 1.07–1.81). There was a significant positive linear trend in all-cause mortality across incremental levels of cumRHR (*P trend* < 0.05 for all models). When regarding cumRHR as a continuous variable, the results were similar. In model 4, each 1-SD [46.78 (beats/min * year)] increase in cumRHR was associated with a 37% increased risk of mortality (adjusted HR: 1.37, 95% CI: 1.23–1.52).

We also investigated interactions between sex and cumRHR. Table 3 displays the hazard ratios (95% CI) for all-cause mortality stratified by sex. Similar results were obtained both for men and women when treating cumRHR as either a continuous or categorical variable. Every 1-SD of cumRHR was associated with a 1.35-fold (95% CI: 1.21–1.51) increased risk of mortality for men and 1.62-fold (95% CI: 1.08–2.44) increased risk of mortality for women. Men in the highest quartile of cumRHR had a 1.40-fold (95% CI: 1.07–1.84) greater risk of all-cause mortality versus the lowest cumRHR quartile. However, the association was not statistically significant among women. The trend was statistically significant only in men (*P trend* = 0.037). There was no significant interaction between cumRHR and sex in relation to all-cause mortality as either a continuous or categorical variable (*P* interaction for both > 0.1).

**Figure 3:**
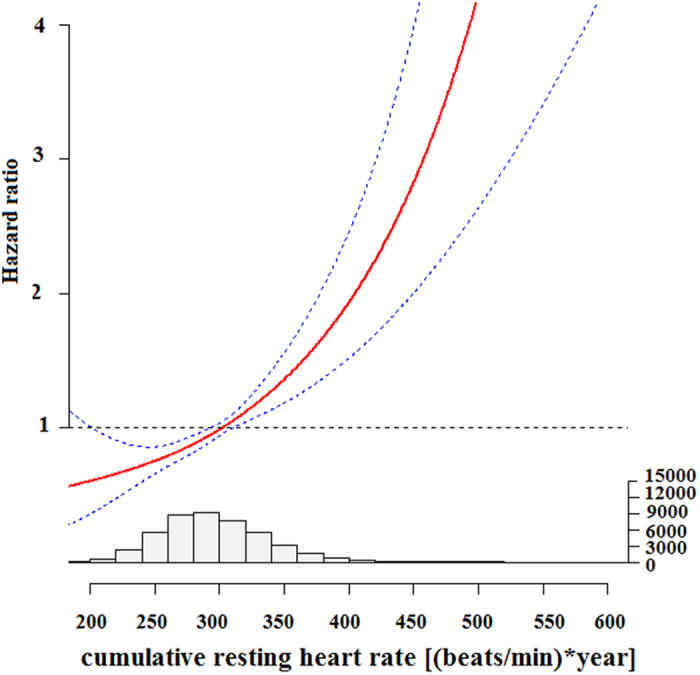
Hazard ratios and 95% confidence intervals for cumRHR with all-cause mortality by using restricted cubic spline regression with three knots with placed at the 25th, 50th, and 75th percentiles of cumRHR, and the red line represented HR and blue lines represented 95% CI. Histogram of cumRHR distribution was shown below graph. Key: CumRHR, cumulative resting heart rate exposure.

The HR (95% CI) for all-cause mortality was estimated using multivariable Cox regression model with the restricted cubic spline for cumRHR. The results are plotted in Fig. 3. The HR for cumRHR with all-cause mortality initially decreased at the lowest quartile, then increased somewhat in the second-lowest quartile, with a higher degree of increase at the upper distributions of cumRHR, producing a J-shaped relationship. There was significant non-linear term for cumRHR (*P* < 0.001), and a threshold value was detected at 300 [(beats/min) * years] as the reference (HR = 1).

### Association with the number of visits with a higher RHR

The baseline characteristics of subjects are summarized by the number of visits with a higher RHR (Supplemental Table 2). Subjects who had a higher RHR status at all 3 study visits were more likely to smoke, drink, be diabetic, and have a higher SBP and TC than individuals who were not higher RHR status. Figure 2(b) shows the Kaplan-Meier morality rate stratified by the number of visits with a higher RHR. There was a progressively increasing risk of morality with the number of visits with a higher RHR, and a dose-response pattern was apparent (log-rank test: χ^2^ = 53.19, *P* < 0.001). In the univariate analysis (model 1), compared with no higher RHR exposure, the hazard ratio for all-cause mortality ranged from 1.37 (95% CI: 1.19–1.59) for 1 study visit with higher RHR to 1.99 (95% CI: 1.53–2.60) for all 3 study visits with higher RHR exposure. After adjustment for all covariates (model 4), the association was reduced, but it still remained statistically significant. Subjects who had a higher RHR at all 3 study visits had a 1.86-fold higher HR (95%CI: 1.33–2.61) for all-cause mortality. There was a significant positive linear trend with the number of visits with a higher RHR (all model *P trend* < 0.001).

Subgroup analysis was followed by stratification of the population by sex, where women who had a higher RHR exposure at all 3 study visits were at greater risk for all-cause mortality (adjusted HR: 3.39, 95% CI: 1.14–10.08) compared with women without higher RHR exposure. And the adjusted HR was 1.72 (95% CI: 1.22–2.49) was fund in men. A positive linear association between the number of visits with a higher RHR and the risk of all-cause mortality was found both in the men and women (*P trend* < 0.001). The relationship between the number of visits with a higher RHR and death was similar regardless of sex (*P* for interaction = 0.18).

### Sensitivity analysis

We found that the results were robust after considering the influence of each subject’s visits time interval. In the fully adjusted model, each 1-SD change in time-weighted cumRHR was associated with 1.22-fold greater risk (95% CI: 1.15–1.30) of all-cause mortality. Compared with participants in the lowest quantile, those subjects in the highest quantile of time-weighted cumRHR had a 77% (HR, 1.77, 95% CI: 1.41–2.21) increased risk of mortality (Supplemental Table 3). Hazard ratios (95% CI) for all-cause mortality according to baseline resting heart rate are displayed in Supplemental Table 4. We found that subjects in the highest cumRHR quartile had a 37% (adjusted HR: 1.37, 95% CI: 1.23–1.52) increased risk of mortality compared with subjects in the lowest cumRHR quartile. Those subjects in the highest baseline RHR quartile also had a 23% (adjusted HR: 1.23, 95% CI: 1.03–1.48) increased risk when using baseline RHR.

## Discussion

In this study of 47,311 subjects from the Kailuan cohort study without a history of myocardial infarction and atrial fibrillation who were followed for 4.06 years, we confirmed that not only does an elevated cumRHR increase the risk of mortality but also the number of visits with a higher RHR increase the risk of mortality in a dose-responsive manner. The association remained highly significant even after adjusting for conventional risk factors and baseline RHR. Importantly, we found that cumRHR had a J-shaped relationship with all-cause mortality.

Our findings were consistent with the results of large population-based epidemiological studies showing that elevated RHR was associated with an increased risk of all-cause mortality^19^,^24^,^25^. However, in most studies, RHR was measured only once at baseline, and comparisons were made using the highest quartile versus the lowest quartile, which would result in bias when estimating the association. Recent studies have used multiple RHR measurements as a time-varying or time-dependent covariate to predict all-cause mortality^6^,^17^,^18^,^19^,^24^. For example, Hartaigh *et al*.^17^ found that each 10 beats/min increment in RHR over the course of six years increased the risk of death by 33% (HR: 1.33, 95% CI: 1.26–1.40) among older adults. Paul *et al*.^26^ found that hypertensive patients with a consistently high RHR over time had a 78% (HR: 1.78, 95% CI: 1.31–2.41) increased risk of all-cause mortality. In our study, we evaluated the cumulative exposure to RHR over time derived from multiple measurements to predict all-cause mortality, which may be a better measure to quantify the relationship of RHR with the risk of death. Summary measures of cumulative exposure that capture both the duration and intensity of RHR could be a more accurate estimate of the effects of RHR over several decades^23^. Both cumRHR exposure and the number of visits with a higher RHR were independent risk factors for all-cause mortality. Our results can extend the association between RHR and death and highlight the importance of cumulative exposure to higher RHR with respect to all-cause mortality.

There are few data assessing the association between cumulative exposure to higher RHR and all-cause mortality. However, cumulative exposure to elevate cholesterol and blood pressure associated with the risk of adverse health outcomes have been reported^21^,^22^,^23^. Navar-Boggan *et al*.^27^ suggested that the risk of CHD increases as the number of individuals exposed to hyperlipidaemia increases in a dose-dependent pattern. Yaffe *et al*.^28^ found that cumulative exposure to cardiovascular risk factors from early to middle adulthood was associated with worse cognition in midlife. Similarly, our study showed that the hazard ratio for subjects who had a higher RHR (≥80 beats/min) at all 3 study visits was 1.86 (95% CI: 1.33–2.61) compared with subject who did not have a higher RHR exposure. A positive linear relationship was found between the number of visits with a higher RHR and all-cause mortality. The Paris Prospective Study found that subjects with increased RHR over 5 years had a 19% increased risk of mortality compared with subjects whose RHR was unchanged^29^, which was in agreement with our findings.

Our findings align with previous studies that reported a J-shaped relationship for baseline RHR with all-cause mortality^30^,^31^. Similarly, we found that cumRHR also demonstrated a J-shaped relationship with increased risk of mortality.

The mechanisms by which cumRHR increases the risk of mortality remain uncertain. A higher RHR is a marker of autonomic nervous system function and is associated with the development of inflammation, atherosclerosis progression, myocardial ischaemia and ventricular arrhythmias^32^. Long-term cumulative exposure to higher RHR may increase the risk of atherosclerosis^33^, hyperinsulinaemia^34^ and hypertension^35^, which can be lead to the development of chronic diseases and death after long-term exposure. We found that elevated cumRHR and the number of visits with a higher RHR were associated with all-cause mortality both in men and women after adjustment for CVD risk factors and baseline RHR. There was no evidence of interaction between sex and cumRHR or the number of higher visits with a higher RHR in association with all-cause mortality. This result was in-line with the findings from the Copenhagen City Heart Study, which similarly found no gender relationship between RHR and mortality^19^.

This study has several strengths, including its large population sample with approximately 4 years of follow-up data. In addition, the RHR was repeatedly measured, and we were able to calculate the cumRHR and the number of visits with a higher RHR, which could more accurately capture long-term effects. Assessment of cumulative exposure could be especially useful for a younger adult with a normal RHR and could confer additional information beyond a single measured RHR at baseline. However, one of the limitations of our study was the unbalanced distribution of gender in the Kailuan cohort study, and most of the participants were male coal miners. Demographic characteristics and cardiac risk factors may differ; therefore, the findings may not be generalised directly to the general Chinese population. In addition, whether this association with all-cause mortality for an overall cohort is causal cannot be determined from this observational study design. Although we carefully adjusted for potential CVD risks factors and baseline RHR, the possibility of residual confounding still remained. The use of cardio-protective medications should also be noted. Subjects who reported using beta-blockers from the baseline to the third study visit were excluded from the current analysis. Information on anti-arrhythmia drugs as well as medications for asthma or COPD was not recorded. Some of the drugs in these classes of medication are related to RHR. Thus, the relationship between cumRHR and the number of visits with a higher RHR and all-cause mortality needs to be validated in other races/ethnic populations.

## Conclusions

We conclude that cumulative exposure to a higher RHR is associated with an increased risk of all-cause mortality in the general population. RHR is a simple clinical measurement, and cumRHR has important prognostic value for predicting all-cause mortality.

## Methods

### Study population

Data were derived from the Kailuan cohort study, a large, observational, prospective and population-based cohort study that was carried out from June 2006 to October 2007 with an enrolment of 101,510 men and women (referred to as the “original cohort”) in Tangshan city in northern China^36^,^37^. The design, methods, rationale and examination details of the Kailuan cohort study were previously published elsewhere^38^. Briefly, all of the participants were visited approximately every two years to obtain information via face-to-face interviews with medical staff and trained research nurses^38^,^39^. Information on routine medical examinations, which included physical examination, electrocardiography and routine blood, urine, and biochemical tests were collected every two years from 11 hospitals^38^. For our investigation, participants were eligible if they attended two consecutive examinations between the second (2008 to 2009) and the third (2010 to 2011) examinations. The exclusion criteria were as follows: history of myocardial infarction or atrial fibrillation, use of heart rate-lowering medications (β-blockers, etc.), or incomplete data on RHR at any of the examinations. A flowchart of the present cohort study is shown below (Fig. 1). All of the participants provided written informed consent, and the study protocol was approved by the Ethics Committee of both Kailuan General Hospital and Beijing Tiantan Hospital and in accordance with the standards set forth by the Declaration of Helsinki. All methods were in-line with approved guidelines.

### Assessment of cumRHR

RHR was measured via a 12-lead electrocardiogram at baseline and at years 2 and 4 with participants resting in the supine position for at least 5 minutes, as previously reported^35^. We calculated the cumulative exposure of RHR (cumRHR) for each subject from the baseline to the third examination [(beats/min) * year] to evaluate long-term exposure to RHR levels. The cumRHR was defined as the summed average RHR for each pair of consecutive examinations multiplied by the time interval between two consecutive examinations in years in reference to the cumSBP^21^:


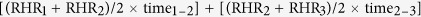


where RHR_1_, RHR_2_, and RHR_3_ indicate the RHR at the baseline, second and third examinations, and time_1–2_ and time_2–3_ indicate the participant-specific time intervals between consecutive examinations in years. The means of time_1–2_ and time_2–3_ were 2.08 years and 1.97 years.

### The number of visits with a higher RHR

According to the Aerobics Center Longitudinal Study (ACLS), adults with a higher RHR (≥80 beats/min) experience an increased risk of all-cause mortality^24^. Therefore, in the current analysis, subjects with a RHR ≥80 beats/min at each study visit were identified to be in the higher RHR status group. The number of visits with a higher RHR was defined as a score ranging from 0 (never had higher RHR) to 3 (had a higher RHR at all three study visits). The cumulative risk of a higher RHR was quantified, and participants were stratified according to the number of visits with a higher RHR: never, at 1 study visit, at 2 study visits and at all 3 study visits.

### Assessment of potential confounding variables

A detailed face-to-face interview, physical examination, anthropometry, and medical history were collected at each examination, as described previously^40^. Data on baseline variables including age, sex, smoking habits, drinking status and physical activity were ascertained from a standard questionnaire^38^. Body mass index (BMI) was calculated as weight in kilograms divided by height in metres squared. Blood pressure was measured with a standard mercury sphygmomanometer by a physician or trained nurse. Blood samples were biochemically measured for serum total cholesterol, low density lipoprotein cholesterol (LDL-C), high-density lipoprotein cholesterol (HDL-C) and fasting blood glucose (FBG)^38^. Hypertension was defined as self-reported use of antihypertensive medication, history of hypertension, systolic blood pressure ≥140 mmHg or diastolic blood pressure ≥90 mmHg. Diabetes mellitus was defined as a fasting blood glucose ≥7.0 mmol/L, taking oral hypoglycaemic agents or insulin or having a self-reported physician diagnosis. Dyslipidaemia was diagnosed as serum total cholesterol ≥5.17 mmol/L, LDL cholesterol ≥3.62 mmol/L, or HDL cholesterol ≤1.04 mmol/L, or using antihyperlipidaemic medications, or self-reported history of dyslipidaemia. History of stroke and cancer was ascertained by self-reported physician diagnosis.

### Outcome

The primary outcome of this study was all-cause mortality. Follow-up began at the third study visit. Participants underwent a clinic examination every two years, and any fatal events were collected through review of death certificates from the provincial vital statistics offices, hospital records, medical insurance data, and interviews with next of kin, relatives, or eyewitnesses, where such undertakings were possible. Vital status was determined by the review committee by December 31, 2014.

### Statistical analysis

The cumRHR was considered both as a continuous and categorical variable. According to the distribution of cumRHR in the general population, cumRHR was categorised into four groups based on quartile: Q1 group, <267.90 (beats/min) * year; Q2 group, 267.90–292.84 (beats/min) * year; Q3 group, 292.85–322.84 (beats/min) * year; Q4 group, 18≥322.85 (beats/min) * year. The baseline characteristics of the study participants were first described according to the cumRHR quartile. Continuous variables were reported as the mean ± standard derivation (SD), and categorical data were summarized using frequency and percentage. Analysis of variance test (ANOVA) or the Kruskal-Wallis test was conducted for continuous variables, and the χ^2^ test was used for categorical variables for global comparisons between the four cumRHR groups.

First, the mortality rate per 10,000 person-years of follow-up was calculated for each quartile of cumRHR and the number of visits with a higher RHR. In addition, analysis of the cumulative mortality rate was performed by the Kaplan-Meier method across cumRHR quartiles and the number of visits with a higher RHR. The log-rank test was used to compare cumulative mortality rate curves. Lastly, Cox proportional hazards model was used to separately estimate the association between cumRHR categorically (first quartile as the reference category) or the number of visits with a higher RHR (no exposure as the reference) and all-cause mortality. Hazard ratios (HR) and 95% confidence intervals (CI) was calculated. The assumption of proportionality was tested by plotting scaled Schoenfeld residuals^41^. Several multivariable Cox proportional hazard models were built to adjust for the effects of confounding covariates: (1) Model 1: a univariate analysis; (2) Model 2: adjusted for age, sex, time_1–2_ and time_2–3_. (3) Model 3: using Model 2 and adding mean systolic blood pressure, smoking, drinking, physical activity, body mass index, hypertension, diabetes mellitus, hyperlipidaemia, and history of stroke and cancer. (4) Model 4: adjusted for the traditional risk factors in Model 3 and adding baseline RHR. We also assessed the relationship between cumRHR when treated as a continuous variable and all-cause mortality per standard deviation (SD) change in the above four models. Subgroup analysis was performed by stratifying the study participants according to sex. Thus, an interaction term for sex and cumRHR or the number of visits with a higher RHR was included in the final model. Further analysis to explore the nonlinear relationships between cumRHR as a continuous variable and all-cause mortality, we used restricted cubic splines with 3 knots at the 25^th^, 50^th^, and 75^th^ percentiles of the cumRHR distribution^42^.

Sensitivity analysis was conducted as follows. First, because the time interval was related to the calculation of cumRHR exposure for each subject, a time-weighted cumRHR was calculated as follows: [(RHR_1_ + RHR_2_)/2 × time_1–2_] + [(RHR_2_ + RHR_3_)/2 × time_2–3_]/(time_1–2_ + time_2–3_). Second, to compare with our results with the baseline RHR, we assessed the relationship between baseline RHR and all-cause mortality.

All analyses were performed using SPSS version 22 (IBM, Armonk, New York, USA), except for the analysis of restricted cubic splines, which was performed with the statistical package R version 3.1.2 (https://www.r-project.org/). All tests were two-sided, and a *P* value of < 0.05 was considered to be statistically significant.

## Additional Information

**How to cite this article**: Zhao, Q. *et al*. Cumulative Resting Heart Rate Exposure and Risk of All-Cause Mortality: Results from the Kailuan Cohort Study. *Sci. Rep.* **7**, 40212; doi: 10.1038/srep40212 (2017).

**Publisher's note:** Springer Nature remains neutral with regard to jurisdictional claims in published maps and institutional affiliations.

## Supplementary Material

Supplementary Information

## Figures and Tables

**Table 1:** Baseline characteristics of 47,311 men and women by cumulative resting heart rate quartiles.

Characteristic	Total	Quartiles of cumulative resting heart rate [(beats/min) * year]				*P*
		Q1 (<267.90)	Q2 (267.90–292.84)	Q3 (292.85–322.84)	Q4 (≥322.85)	
No. of subjects	47311	11828	11832	11823	11828	
Age, year	48.70 ± 11.68	46.09 ± 10.58	47.07 ± 11.37	49.70 ± 11.68	51.92 ± 12.15	<0.001 *
Men sex, n (%)	37010 (78.2)	9185 (77.7)	9276 (78.4)	9245 (78.2)	9304 (78.7)	0.285#
Current smoker, n (%)	14480 (30.6)	3381 (28.6)	3703 (31.3)	3670 (31.0)	3724 (31.5)	<0.001#
Current drinker, n (%)	8471 (17.9)	1868 (15.8)	2118 (17.9)	2206 (18.7)	2279 (19.3)	<0.001#
Physical activity, n (%)	6424 (13.6)	1279 (10.8)	1456 (12.3)	1683 (14.2)	2006 (17.0)	<0.001#
SBP, mmHg	128.61 ± 19.90	124.55 ± 18.64	127.21 ± 19.09	129.46 ± 19.77	133.21 ± 20.99	<0.001*
DBP, mmHg	82.73 ± 11.40	80.87 ± 10.86	82.20 ± 11.08	83.11 ± 11.35	84.74 ± 11.94	<0.001*
RHR, beats/min	73.41 ± 9.83	67.74 ± 7.35	71.86 ± 7.67	74.41 ± 8.87	79.63 ± 11.00	<0.001*
BMI, kg/m^2^	25.06 ± 3.47	24.95 ± 3.37	25.12 ± 3.46	25.06 ± 3.49	25.09 ± 3.55	0.002*
FBG, mmol/L	5.40 ± 1.54	5.18 ± 1.25	5.30 ± 1.36	5.46 ± 1.56	5.67 ± 1.86	<0.001*
TC, mmol/L	4.94 ± 1.14	4.83 ± 1.11	4.88 ± 1.14	4.95 ± 1.14	5.09 ± 1.14	<0.001*
HDL-C, mmol/L	1.56 ± 0.40	1.56 ± 0.39	1.56 ± 0.40	1.55 ± 0.40	1.56 ± 0.41	0.462*
LDL-C, mmol/L	2.30 ± 0.92	2.22 ± 0.90	2.30 ± 0.92	2.31 ± 0.92	2.38 ± 0.94	<0.001*
Hypertension, n (%)	19170 (40.5)	3890 (32.9)	4461 (37.7)	4963 (42.0)	5860 (49.5)	<0.001#
Diabetes mellitus, n (%)	461 (10.2)	842 (7.1)	1072 (9.1)	1292 (10.9)	1610 (13.6)	<0.001#
Hyperlipidemia, n (%)	22045 (46.6)	4901 (41.1)	5332 (45.1)	5647 (47.8)	6165 (52.1)	<0.001#
Stroke, n (%)	628 (1.3)	88 (0.7)	113 (1.0)	180 (1.5)	247 (2.1)	<0.001#
Cancer, n (%)	126 (0.3)	20 (0.2)	26 (0.2)	33 (0.3)	47 (0.4)	0.002#

Data shown as mean ± SD or frequency (percentage).

Key: SBP, systolic blood pressure; DBP, Diastolic blood pressure; RHR, resting heart rate; BMI, body mass index; FBG, fasting blood glucose; TC, total cholesterol; HDL-C, higher-density lipoprotein cholesterol; LDL-C, low-density lipoprotein cholesterol.

^*^Analysis of variance test (ANOVA) or Kruskal-Wallis test used for continuous variables.

^#^χ^2^ test used for categorical variables.

**Table 2:** Hazard ratios (95% CI) for all-cause mortality according to the cumulative resting heart rate or the number of visits with a higher RHR.

cumRHR (bmp*year)	Participants (No.)	Death (No.)	Mortality rate*	Hazard ratio (95% CI)#			
				Model 1†	Model 2‡	Model 3§	Model 4||
Change per SD (46.78)	47311	1025	54.44	1.35 (1.29–1.42)	1.38 (1.28–1.49)	1.27 (1.17–1.37)	1.37 (1.23–1.52)
Quartiles							
Q1:<267.90	11828	176	35.60	1 (Reference)	1 (Reference)	1 (Reference)	1 (Reference)
Q2:267.90–292.84	11832	203	42.05	1.18 (0.96–1.44)	1.08 (0.88–1.33)	1.08 (0.87–1.35)	1.07 (0.86–1.34)
Q3:292.85–322.84	11823	246	52.94	1.47 (1.21–1.79)	1.12 (0.91–1.38)	1.09 (0.88–1.37)	1.09 (0.87–1.36)
Q4:≥322.85	11828	400	90.91	2.54 (2.12–3.04)	1.70 (1.38–2.10)	1.42 (1.13–1.78)	1.39 (1.07–1.81)
*P* for trend				<0.001	<0.001	0.004	0.027
The number of visits with a higher RHR							
Never	29690	542	44.34	1 (Reference)	1 (Reference)	1 (Reference)	1 (Reference)
At 1 study visit	11105	280	63.35	1.37 (1.19–1.59)	1.43 (1.24–1.65)	1.31 (1.12–1.52)	1.35 (1.15–1.59)
At 2 study visits	4816	142	74.27	1.63 (1.35–1.96)	1.78 (1.48–2.15)	1.52 (1.25–1.85)	1.62 (1.30–2.02)
At 3 study visits	1700	61	91.30	1.99 (1.53–2.60)	2.07 (1.59–2.70)	1.66 (1.25–2.21)	1.86 (1.33–2.61)
*P* for trend				<0.001	<0.001	<0.001	<0.001

^#^Hazard ratio (95% CI) was calculated from Cox models.

^*^Mortality rate per 10, 000 person-years.

^†^Univariate analysis.

^‡^Adjusted for age, sex, time_1–2_ and time_2–3_.

^§^Adjusted for age, sex, time_1–2_, time_2–3_, mean systolic blood pressure, smoking, drinking, physical activity, body mass index, hypertension, diabetes mellitus, hyperlipidemia, stroke and cancer.

^||^Adjusted for risks factor in model 3 plus baseline resting heart rate.

**Table 3:** Hazard ratios (95% CI) of all-cause mortality stratified analysis by sex.

	Adjusted Hazard ratio* (95% CI)		*P* for interaction
	Men (n = 37010)	Women (n = 10301)	
Cumulative Resting Heart Rate			
Change per SD	1.35 (1.21–1.51)	1.62 (1.08–2.44)	0.857
Quartiles			0.902
Q1:<267.90	1 (Reference)	1 (Reference)	
Q2:267.90–292.84	1.09 (0.87–1.37)	0.94 (0.43–2.07)	
Q3:292.85–322.84	1.10 (0.86–1.39)	0.95 (0.41–2.19)	
Q4:≥322.85	1.40 (1.07–1.84)	1.28 (0.47–3.48)	
*P* for trend	0.037	0.830	
The number of visits with a higher RHR			0.180
Never	1 (Reference)	1 (Reference)	
At 1 study visit	1.37 (1.16–1.62)	1.11 (0.59–2.08)	
At 2 study visits	1.57 (1.25–1.97)	2.54 (1.20–5.40)	
At 3 study visits	1.72 (1.22–2.49)	3.39 (1.14–10.08)	
*P* for trend	<0.001	0.038	

^*^Adjusted for age, sex, time_1–2_, time_2–3_, mean systolic blood pressure, smoking, drinking, physical activity, body mass index, hypertension, diabetes mellitus, hyperlipidemia, stroke, cancer and baseline resting heart rate by multivariate Cox models.

## References

[b1] Wang A. X. . Resting Heart Rate and Risk of Cardiovascular Diseases and All-Cause Death: The Kailuan Study. Plos One 9, e110985, doi: ARTN e11098510.1371/journal.pone.0110985 (2014). 25343354 10.1371/journal.pone.0110985 PMC4208799

[b2] Cubbon R. M. . Ambulatory heart rate range predicts mode-specific mortality and hospitalisation in chronic heart failure. Heart 102, 223–229, doi: 10.1136/heartjnl-2015-308428 (2016). 26674986 PMC4752612

[b3] Lupon J. . Aging and Heart Rate in Heart Failure: Clinical Implications for Long-term Mortality. Mayo Clin Proc 90, 765–772, doi: 10.1016/j.mayocp.2015.02.019 (2015). 26046411

[b4] Laskey W. K. . Heart rate at hospital discharge in patients with heart failure is associated with mortality and rehospitalization. J Am Heart Assoc 4, e001626, doi: 10.1161/JAHA.114.001626 (2015). 25904590 PMC4579947

[b5] Lancellotti P., Ancion A., Magne J., Ferro G. & Pierard L. A. Elevated heart rate at 24–36 h after admission and in-hospital mortality in acute in non-arrhythmic heart failure. Int J Cardiol 182, 426–430, doi: 10.1016/j.ijcard.2015.01.027 (2015). 25596471

[b6] Steinberg B. A. . Increased Heart Rate Is Associated With Higher Mortality in Patients With Atrial Fibrillation (AF): Results From the Outcomes Registry for Better Informed Treatment of AF (ORBIT-AF). J Am Heart Assoc 4, e002031, doi: 10.1161/JAHA.115.002031 (2015). 26370445 PMC4599492

[b7] Gabet A., Juilliere Y., Lamarche-Vadel A., Vernay M. & Olie V. National trends in rate of patients hospitalized for heart failure and heart failure mortality in France, 2000–2012. Eur J Heart Fail 17, 583–590, doi: 10.1002/ejhf.284 (2015). 25950872

[b8] Ford I. . Top ten risk factors for morbidity and mortality in patients with chronic systolic heart failure and elevated heart rate: The SHIFT Risk Model. Int J Cardiol 184, 163–169, doi: 10.1016/j.ijcard.2015.02.001 (2015). 25703424

[b9] Kallmunzer B. . Impact of heart rate dynamics on mortality in the early phase after ischemic stroke: a prospective observational trial. J Stroke Cerebrovasc Dis 24, 946–951, doi: 10.1016/j.jstrokecerebrovasdis.2014.12.009 (2015). 25804569

[b10] Bohm M. . Impact of resting heart rate on mortality, disability and cognitive decline in patients after ischaemic stroke. Eur Heart J 33, 2804–2812, doi: 10.1093/eurheartj/ehs250 (2012). 22922507

[b11] Jorgensen R. M. . Heart Rate Variability Density Analysis (Dyx) and Prediction of Long-Term Mortality after Acute Myocardial Infarction. Ann Noninvasive Electrocardiol 21, 60–68, doi: 10.1111/anec.12297 (2015). 26262922 PMC6931517

[b12] Warnier M. J., Rutten F. H., de Boer A., Hoes A. W. & De Bruin M. L. Resting heart rate is a risk factor for mortality in chronic obstructive pulmonary disease, but not for exacerbations or pneumonia. Plos One 9, e105152, doi: 10.1371/journal.pone.0105152 (2014). 25157876 PMC4144884

[b13] Hillis G. S. . Resting heart rate and the risk of death and cardiovascular complications in patients with type 2 diabetes mellitus. Diabetologia 55, 1283–1290, doi: 10.1007/s00125-012-2471-y (2012). 22286552 PMC4170780

[b14] Li Y. Association between resting heart rate and cardiovascular mortality: evidence from a meta-analysis of prospective studies. Int J Clin Exp Med 8, 15329–15339 (2015). 26629022 PMC4658911

[b15] Zhang D., Shen X. & Qi X. Resting heart rate and all-cause and cardiovascular mortality in the general population: a meta-analysis. CMAJ 188, e53–63, doi: 10.1503/cmaj.150535 (2015). 26598376 PMC4754196

[b16] Xu S. . Estimating the effects of time-varying exposures in observational studies using Cox models with stabilized weights adjustment. Pharmacoepidemiol Drug Saf 23, 812–818, doi: 10.1002/pds.3601 (2014). 24596337 PMC4351798

[b17] Hartaigh B. . Elevations in time-varying resting heart rate predict subsequent all-cause mortality in older adults. Eur J Prev Cardiol 22, 527–534, doi: 10.1177/2047487313519932 (2015). 24445263 PMC4156557

[b18] Jennifer, E., Ho M. . Long-term Cardiovascular Risks Associated With an Elevated Heart Rate: The Framingham Heart Study. Journal of the American Heart Association 3, e000668, doi: 10.1161/JAHA.113.000668 (2014). 24811610 PMC4309047

[b19] Jensen M. T., Marott J. L. & Jensen G. B. Elevated resting heart rate is associated with greater risk of cardiovascular and all-cause mortality in current and former smokers. Int J Cardiol 151, 148–154, doi: 10.1016/j.ijcard.2010.05.003 (2011). 20605243

[b20] de Vocht F., Burstyn I. & Sanguanchaiyakrit N. Rethinking cumulative exposure in epidemiology, again. Journal of Exposure Science and Environmental Epidemiology 25, 467–473 (2015). 25138292 10.1038/jes.2014.58

[b21] Zemaitis P. . Cumulative systolic BP and changes in urine albumin-to-creatinine ratios in nondiabetic participants of the multi-ethnic study of atherosclerosis. Clin J Am Soc Nephrol 9, 1922–1929, doi: 10.2215/CJN.02450314 (2014). 25200476 PMC4220754

[b22] Kishi S. . Cumulative Blood Pressure in Early Adulthood and Cardiac Dysfunction in Middle Age: The CARDIA Study. J Am Coll Cardiol 65, 2679–2687, doi: 10.1016/j.jacc.2015.04.042 (2015). 26112189

[b23] Nikpour M., Urowitz M. B., Ibanez D., Harvey P. J. & Gladman D. D. Importance of cumulative exposure to elevated cholesterol and blood pressure in development of atherosclerotic coronary artery disease in systemic lupus erythematosus: a prospective proof-of-concept cohort study. Arthritis Research & Therapy 13, 1–14 (2011). 10.1186/ar3473 PMC3308087 21955652

[b24] Saxena A. . Protective role of resting heart rate on all-cause and cardiovascular disease mortality. Mayo Clin Proc 88, 1420–1426, doi: 10.1016/j.mayocp.2013.09.011 (2013). 24290115 PMC3908776

[b25] Woodward M. . The association between resting heart rate, cardiovascular disease and mortality: evidence from 112,680 men and women in 12 cohorts. Eur J Prev Cardiol 21, 719–726, doi: 10.1177/2047487312452501 (2014). 22718796

[b26] Paul L. . Resting heart rate pattern during follow-up and mortality in hypertensive patients. Hypertension 55, 567–574, doi: 10.1161/HYPERTENSIONAHA.109.144808 (2010). 20038750

[b27] Yaffe K. . Early adult to midlife cardiovascular risk factors and cognitive function. Circulation 129, 1560–1567, doi: 10.1161/CIRCULATIONAHA.113.004798 (2014). 24687777 PMC4700881

[b28] Navar-Boggan A. M. . Hyperlipidemia in early adulthood increases long-term risk of coronary heart disease. Circulation 131, 451–458, doi: 10.1161/CIRCULATIONAHA.114.012477 (2015). 25623155 PMC4370230

[b29] X. J. . Relation of heart rate at rest and longterm (20 years) death rate in initially healthy middle-aged men, doi: 10.1016/j.amjcard.2008.08.071. 19121452

[b30] Custodis F. . Resting heart rate is an independent predictor of all-cause mortality in the middle aged general population. Clin Res Cardiol 1–12, doi: 10.1007/s00392-015-0956-7 (2016). 26803646

[b31] Kuzuya M., Enoki H., Iwata M., Hasegawa J. & Hirakawa Y. J-shaped relationship between resting pulse rate and all-cause mortality in community-dwelling older people with disabilities. J Am Geriatr Soc 56, 367–368, doi: 10.1111/j.1532-5415.2007.01512.x (2008). 18251825

[b32] Caetano J. & Alves J. D. Heart rate and cardiovascular protection. European Journal of Internal Medicine 30, 217–222 (2015). 10.1016/j.ejim.2015.02.009 25704330

[b33] Whelton S. P. . Association of resting heart rate with carotid and aortic arterial stiffness: multi-ethnic study of atherosclerosis. Hypertension 62, 477–484 (2013). 23836802 10.1161/HYPERTENSIONAHA.113.01605 PMC3838105

[b34] Wang L. . Resting heart rate and the risk of developing impaired fasting glucose and diabetes: the Kailuan prospective study. Int J Epidemiol 44, 689–699, doi: 10.1093/ije/dyv079 (2015). 26002923 PMC4553707

[b35] Wang A. X. . Resting heart rate and risk of hypertension: results of the Kailuan cohort study. J Hypertens 32, 1600–1605, doi: 10.1097/Hjh.0000000000000230 (2014). 24879491

[b36] Wang A. X. . Hypertriglyceridemic waist phenotype and risk of cardiovascular diseases in China: Results from the Kailuan Study. International Journal of Cardiology 174, 106–109, doi: 10.1016/j.ijcard.2014.03.177 (2014). 24745860

[b37] Li Z. . Impact of proteinuria and glomerular filtration rate on risk of ischaemic and intracerebral hemorrhagic stroke: a result from the Kailuan study. Eur J Neurol 22, 355–360, doi: 10.1111/ene.12580 (2015). 25346321

[b38] Wang A. X. . Measures of Adiposity and Risk of Stroke in China: A Result from the Kailuan Study. Plos One 8, e61665, doi: ARTN e6166510.1371/journal.pone.0061665 (2013). 23613897 10.1371/journal.pone.0061665 PMC3629147

[b39] Peng M. . Long-term alcohol consumption is an independent risk factor of hypertension development in northern China: evidence from Kailuan study. J Hypertens 31, 2342–2347, doi: 10.1097/HJH.0b013e3283653999 (2013). 24029874

[b40] Zhang Q., Zhang S. F., Bian L. H. & Zhao X. Q. Ideal Cardiovascular Health Metrics on Risk of Ischemic and Intracerebral Hemorrhagic Stroke: A Result from Kailuan Study. Stroke 44, 2451–2456 (2013). 23868276 10.1161/STROKEAHA.113.678839

[b41] Xue X. . Testing the proportional hazards assumption in case-cohort analysis. BMC Med Res Methodol 13, 1–10, doi: 10.1186/1471-2288-13-88 (2013). 23834739 PMC3710085

[b42] Rutherford M. J., Crowther M. J. & Lambert P. C. The use of restricted cubic splines to approximate complex hazard functions in the analysis of time-to-event data: a simulation study. Journal of Statistical Computation and Simulation 85, 777–793 (2015).

