# Supplementary material for: Cumulative Resting Heart Rate Exposure and Risk of All-Cause Mortality: Results from the Kailuan Cohort Study
Source: Sci Rep. 2017 Jan 9;7:40212. doi: 10.1038/srep40212 (PMC5220288; doi:10.1038/srep40212)
Supplement: Supplementary Information [file srep40212-s1.pdf]

Full title: Cumulative Resting Heart Rate Exposure and Risk of All-Cause Mortality: Results from the Kailuan Cohort Study

Authors: Quanhui Zhao[1, 2], MD*; Haibin Li[3, 4], MS*; Anxin Wang[3, 4, 5], PhD*; Jin Guo[3, 4], PhD; Junxing Yu, MD[1, 2*]; Yanxia Luo[3, 4], PhD; Shuohua Chen[2], MD; Lixin Tao[3, 4], PhD; Yuqing Li[6], MD; Aiping Li[7], MD; Xiuhua Guo[3, 4], PhD[#] ; Shouling Wu[2#], MD

Author's Affiliation:

[1] Graduate School, North China University of Science and Technology, Tangshan, China.

[2] Department of Cardiology, Kailuan Hospital, North China University of Science and Technology, Tangshan, China.

[3] Department of Epidemiology and Health Statistics, School of Public Health, Capital Medical University, Beijing, China.

[4] Beijing Municipal Key Laboratory of Clinical Epidemiology, Capital Medical University, Beijing, China.

[5] Department of Neurology, Beijing Tiantan Hospital, Capital Medical University, Beijing, China

[6] Department of Rheumatology, Kailuan Hospital, North China University of Science and Technology, Tangshan, China.

[7] Department of Surgery, Kailuan Hospital, North China University of Science and Technology, Tangshan, China.

Supplementary Information

Supplemental Table 1. Baseline characteristics stratified by completed data and missing data on rest heart rate from Kailuan cohort study

Characteristic	Completed data	Missing rest heart rate	*P*
No. of subjects	47311	8930	
Age, year	48.70 ± 11.68	51.78 ± 12.78	<0.001*
Men, n (%)	37010 (78.2)	5929 (66.4)	<0.001#
Current smoker, n (%)	14480 (30.6)	2346 (27.3)	<0.001#
Current drinker, n (%)	8471 (17.9)	1348 (15.7)	<0.001#
Physical activity, n (%)	6424 (13.6)	1333 (16.4)	<0.001#
SBP, mmHg	128.61 ± 19.90	127.03 ± 20.15	<0.001*
DBP, mmHg	82.73 ± 11.40	81.31 ± 11.20	<0.001*
BMI, kg/m^2	25.06 ± 3.47	25.04 ± 3.49	0.678*
FBG, mmol/L	5.40 ± 1.54	5.36 ± 1.56	0.011*
TC, mmol/L	4.94 ± 1.14	4.89 ± 1.10	0.002*
HDL-C, mmol/L	1.56 ± 0.40	1.50 ± 0.36	<0.001*
LDL-C, mmol/L	2.30 ± 0.92	2.25 ± 0.78	<0.001*
Hypertension, n (%)	19174 (40.5)	3334 (37.3)	0.001#
Diabetes mellitus, n (%)	4816 (10.2)	807 (9.0)	0.001#
Hyperlipidemia, n (%)	22045 (46.6)	4072 (45.5)	0.064#
Stroke, n (%)	628 (1.3)	233 (2.6)	<0.001#
Cancer, n (%)	126 (0.3)	37 (0.5)	0.006#

Data shown as mean±SD or frequency (percentage).

Key: SBP, systolic blood pressure; DBP, Diastolic blood pressure; RHR, resting heart rate; BMI, body mass index; FBG, fasting blood glucose; TC, total cholesterol; HDL-C, higher-density lipoprotein cholesterol; LDL-C, low-density lipoprotein cholesterol.

*Analysis of variance test (ANOVA) or Kruskal-Wallis test used for continuous variables;

#χ^2 test used for categorical variables.

Supplemental Table 2. Baseline characteristics of 47,311 men and women stratified by the number of higher RHR status

Characteristic	Total	The number of visits with a higher RHR (≥ 80 beats/min)				*P*
		Never	At 1 study visit	At 2 study visits	At 3 study visits	
No. of subjects	47311	29690	11105	4816	1700	
Age, year	48.70 ± 11.68	49.07 ± 11.59	48.24 ± 11.76	47.75 ± 11.83	47.82 ± 12.10	<0.001*
Men, n (%)	37010 (78.2)	22831 (76.9)	8862 (79.8)	3929 (81.6)	1388 (81.6)	<0.001#
Current smoker, n (%)	14480 (30.6)	8491 (28.6)	3590 (32.3)	1754 (36.4)	645 (37.9)	<0.001#
Current drinker, n (%)	8471 (17.9)	5041 (17.0)	2079 (18.7)	977 (20.3)	374 (22.0)	<0.001#
Physical activity, n (%)	6424 (13.6)	4350 (14.7)	1311 (11.8)	563 (11.7)	200 (11.8)	<0.001#
SBP, mmHg	126.81 ± 19.90	126.73 ± 19.14	130.24 ± 20.19	133.63 ± 21.21	136.45 ± 21.70	<0.001*
DBP, mmHg	82.73 ± 11.40	81.57 ± 10.87	83.87 ± 11.63	85.84 ± 12.43	86.75 ± 12.53	<0.001*
RHR, beats/min	73.41 ± 9.83	69.43 ± 6.56	76.89 ± 9.23	83.11 ± 10.38	92.71 ± 9.08	<0.001*
BMI, kg/m^2	25.06 ± 3.47	25.03 ± 3.04	25.05 ± 3.48	25.23 ± 3.73	25.05 ± 3.70	0.030*
FBG, mmol/L	5.40 ± 1.54	5.27 ± 1.35	5.50 ± 1.64	5.79 ± 1.93	6.02 ± 2.09	<0.001*
TC, mmol/L	4.94 ± 1.14	4.88 ± 1.23	4.98 ± 1.12	5.11 ± 1.18	5.15 ± 1.20	<0.001*
HDL-C, mmol/L	1.56 ± 0.40	1.55 ± 0.40	1.56 ± 0.40	1.58 ± 0.41	1.59 ± 0.42	<0.001*
LDL-C, mmol/L	2.30 ± 0.92	2.26 ± 0.93	2.34 ± 0.90	2.46 ± 0.93	2.44 ± 0.90	<0.001*
Hypertension, n (%)	19174 (40.5)	11017 (37.1)	4806 (43.3)	2422 (50.3)	929 (54.6)	<0.001#
Diabetes mellitus, n (%)	4816 (10.2)	2729 (9.2)	1136 (10.2)	660 (13.7)	291 (17.1)	<0.001#
Hyperlipidemia, n (%)	22045 (46.6)	13186 (44.4)	5345 (48.1)	2576 (53.5)	938 (55.2)	<0.001#
Stroke, n (%)	628 (1.3)	397 (1.3)	142 (1.3)	76 (1.6)	13 (0.8)	0.084#
Cancer, n (%)	126 (0.3)	75 (0.3)	18 (0.2)	27 (0.6)	6 (0.4)	<0.001#

Data shown as mean ± SD or frequency (percentage).

Key: SBP, systolic blood pressure; DBP, Diastolic blood pressure; RHR, resting heart rate; BMI, body mass index; FBG, fasting blood glucose; TC, total cholesterol; HDL-C, higher-density lipoprotein cholesterol; LDL-C, low-density lipoprotein cholesterol.

* Analysis of variance test (ANOVA) or Kruskal-Wallis test used for continuous variables;

#χ^2 test used for categorical variables.

Supplemental Table 3. Hazard ratios (95% CI) for all-cause mortality according to time-weighted cumRHR

	Participants	Death	Mortality	Hazard ratio (95% CI)#			
RHR [(beat/min)*year]	(No.)	(No.)	rate*	Model 1†	Model 2‡	Model 3§	Model 4‖
Change per SD (8.05)	47311	1025	54.43	1.20 (1.13-1.27)	1.26 (1.20-1.34)	1.22 (1.15-1.30)	1.30 (1.20-1.41)
Quartiles							
Q1:<68.70	11822	220	47.11	1(Reference)	1(Reference)	1(Reference)	1(Reference)
Q2:68.70-73.20	11829	224	47.34	1.01 (0.84-1.21)	1.17 (0.97-1.41)	1.17 (0.96-1.43)	1.19 (0.97-1.46)
Q3:73.20-78.26	11833	252	53.64	1.14 (0.95-1.36)	1.34 (1.11-1.60)	1.30 (1.08-1.58)	1.34 (1.09-1.64)
Q4:⩾78.26	11827	329	69.89	1.48 (1.25-1.76)	1.80 (1.52-2.14)	1.67 (1.39-2.01)	1.77 (1.41-2.21)
P for trend				<0.001	<0.001	<0.001	<0.001

Hazard ratio (95% CI) was calculated from Cox models

*Mortality rate per 10, 000 person-years

†Univariate analysis

‡Adjusted for age, sex.

§Adjusted for age, sex, mean systolic blood pressure, smoking, drinking, physical activity, body mass index, hypertension, diabetes mellitus, hyperlipidemia, stroke and cancer.

‖Adjusted for risks factor in model 3 plus baseline resting heart rate.

Supplemental Table 4. Hazard ratios (95% CI) for all-cause mortality according to baseline resting heart rate

RHR [(beat/min)*year]	Participants (No.)	Death (No.)	Mortality rate*	Hazard ratio (95% CI)#		
				Model 1†	Model 2‡	Model 3§
Change per SD (9.83)	47311	1025	54.43	1.07 (1.00-1.13)	1.13 (1.07-1.20)	1.11 (1.05-1.18)
Quartiles						
Q1:<68.00	10666	232	55.06	1(Reference)	1(Reference)	1(Reference)
Q2:68.00-72.00	11102	221	49.76	0.91 (0.75-1.09)	1.13 (0.94-1.36)	1.09 (0.89-1.33)
Q3:72.00-79.00	13047	271	52.32	0.93 (0.78-1.11)	1.07 (0.90-1.28)	1.03 (0.89-1.24)
Q4:⩾79.00	12496	301	60.52	1.10 (0.93-1.30)	1.37 (1.16-1.63)	1.23 (1.03-1.48)
P for trend				0.108	0.002	0.093

Hazard ratio (95% CI) was calculated from Cox models

*Mortality rate per 10, 000 person-years

†Univariate analysis

‡Adjusted for age, sex.

§Adjusted for age, sex, mean systolic blood pressure, smoking, drinking, physical activity, body mass index, hypertension, diabetes mellitus, hyperlipidemia, stroke and cancer.
